# Morphogenesis and Topography of the Mesorectal Fascia

**DOI:** 10.3390/jcm15041377

**Published:** 2026-02-10

**Authors:** Iulian Alexandru Dogaru, Adrian Daniel Tulin, Iulian Mirel Slavu, Daniela Elena Gheoca Mutu, Mihaly Enyedi, Răzvan Stănciulescu, Cosmin Marian Panțu, Zoran Florin Filipoiu

**Affiliations:** 1 Doctoral School, “Carol Davila” University of Medicine and Pharmacy, 020021 Bucharest, Romania; iulian-alexandru.dogaru@drd.umfcd.ro; 2 Discipline of Anatomy, Faculty of Medicine, Preclinical Department 2—Morphological Sciences, “Carol Davila” University of Medicine and Pharmacy, 020021 Bucharest, Romania; iulian.slavu@umfcd.ro (I.M.S.); daniela-elena.mutu@umfcd.ro (D.E.G.M.); mihaly.enyedi@umfcd.ro (M.E.); razvan.stanciulescu@umfcd.ro (R.S.); cosmin.pantu@umfcd.ro (C.M.P.); 3 Clinical Department of General Surgery, “Prof. Dr. Agrippa Ionescu” Clinical Emergency Hospital, 011356 Bucharest, Romania; 4 Clinical Department of Plastic and Aesthetic Surgery and Reconstructive Microsurgery, “Prof. Dr. Agrippa Ionescu” Clinical Emergency Hospital, 011356 Bucharest, Romania; 5 Faculty of Medicine, “Carol Davila” University of Medicine and Pharmacy, 020021 Bucharest, Romania; zoran-florin.filipoiu2022@stud.umfcd.ro

**Keywords:** embryonic and fetal development, rectum, hypogastric plexus, mesorectum, total mesorectal excision

## Abstract

**Background**: First described by Carl Toldt in the late 19th century, the mesorectum has since been a topic of anatomical and surgical debate. Its clinical importance was redefined by Heald’s introduction of Total Mesorectal Excision (TME), nowadays the golden standard in oncologic rectal surgery. This study aims to elucidate the embryological development and adult anatomy of the mesorectum and the mesorectal fascia, with a focus on clinically significant relations, particularly the peritoneum, and components of the hypogastric plexuses. **Methods**: We performed anatomical dissections on four 12–15-week-old human fetuses and eight formalin-fixed adult cadavers. In addition, a transverse pelvic section was examined to assess the spatial organization of mesorectal and fascial structures. **Results**: Our findings confirm the presence of a dorsal mesentery at the rectal level during fetal development, illustrating its transformation into the adult mesorectum. We identified the mesorectal contents in the fetus and examined the course and relations of the superior rectal vessels, hypogastric nerves, and pelvic splanchnic nerves, in both fetal and adult specimens. **Conclusions**: The observed fetal and adult configurations provide a continuous morphological description of the mesorectum and its compartmental organization within the pelvis. This study enhances the understanding of the mesorectum’s embryology, structure, and vital surgical landmarks. By delineating the so-called ‘Holy plane’ of Heald (the natural avascular plane between the mesorectal and presacral fasciae used during total mesorectal excision) and the delicate connective fibers known surgically as ‘angel’s hair’, which become visible when this plane is correctly entered, rectal and presacral fasciae, and neurovascular elements, provides a comprehensive anatomical framework that may inform surgical plane identification and support future clinical investigations into nerve-sparing rectal surgery.

## 1. Introduction

The surgical anatomy of the mesorectum represents a continuously evolving field, bridging descriptive morphology, embryology, and oncologic surgery. In 1885, Frederick Treves first outlined crucial anatomical concepts of the intestinal tube and its peritoneal dependencies, setting the foundation for the understanding of visceral and mesenteric anatomy [1]. Shortly thereafter, at the end of the 19th century, Thoma Ionescu (Thomas Jonnesco) provided the first description of a distinct fascial layer surrounding the rectum, the “rectal sheath”, in a chapter of Poirier’s historical landmark anatomical treatise, ‘Traité d’Anatomie Humaine’, published in 1894 [2]. Ionescu clearly detailed the “rectal sheath”, the fascial envelope surrounding the rectum and its relation to surrounding structures, establishing a crucial basis for subsequent surgical approaches. Wilhelm von Waldeyer later independently referred to the same anatomical entity as the “fascia propria recti”, reinforcing its conceptual significance within the European anatomical literature [3,4]. These findings provided an anatomical framework which is crucial to what is nowadays known as the “mesorectum”, a concept that has since been interpreted in multiple ways, sparking considerable debate regarding its exact anatomical definition, specifically whether it constitutes a true mesentery or a fascial envelope, as well as its boundaries and clinical significance [5].

Richard J. Heald’s revolutionary contribution, the total mesorectal excision (TME), placed the mesorectum in a surgical context that required a thorough anatomical understanding [6]. This subsequently turned the mesorectum into a cornerstone of surgical oncology, significantly improving the prognosis of rectal cancer patients [7]. By establishing a standardized surgical strategy based on dissection in the anatomical and embryological tissue planes, TME underscores the necessity of accurate anatomical knowledge, with an emphasis on the deep fascial structures of the pelvis and the autonomic nervous elements [8].

In this context, a thorough understanding of the embryologic development of the mesorectum and its intricate relationships with the pelvic fascial and nervous structures is particularly valuable. The relationships between the mesorectal fascia, the peritoneum, and the hypogastric plexuses constitute a morphologic matrix, the clarification of which directly impacts the safety and radicality of surgical maneuvers. Despite significant advances in rectal surgery, the literature still presents areas of uncertainty regarding the precise topography and boundaries of the mesorectal sheath, its cranio-caudal continuity, and its demarcation from neighboring nervous structures.

Our study analyzes the embryologic development and adult anatomy of the mesorectum through correlated dissections of human fetal and adult specimens. Emphasis is placed on the transformation of the dorsal mesentery of the rectum, the formation of the mesorectal fascia, and the spatial organization of the pelvis. The analysis follows these structures, documenting their morphology and relationships as observed in situ. We used the term “mesorectal fascia” as the standard term throughout the paper to align with modern surgical nomenclature, whilst regarding “fascia propria recti” and “rectal sheath” as historical synonyms.

## 2. Materials and Methods

Our study was performed on four human fetuses aged 12–15 weeks of gestation and eight formalin-fixed adult cadavers (five males, three females). According to the records of the Department of Anatomy, the specimens had no documented history of major pelvic surgery, pelvic malignancy, or significant pelvic pathology that could alter normal fascial anatomy. Exact ages were not consistently available; however, all specimens represented mature adult donors used for anatomical education. All specimens were obtained and handled by the Discipline of Anatomy, Faculty of Medicine, „Carol Davila” University of Medicine and Pharmacy, Bucharest, Romania, in accordance with the national legislation governing the use of human cadaveric material for educational and research purposes (Law no. 104/27 March 2003, republished). The study was approved by the Ethics Committee of the University (protocol code 21062/1 October 2021).

In fetal specimens, dissection of the abdominal cavity was performed by removing the anterior abdominal wall and selectively excising the abdominopelvic viscera, mainly preserving the rectum, kidneys, ureters and main abdominopelvic vessels, as well as the peritoneum surrounding the pelvic viscera. The rectum and the remnants of the dorsal mesentery were dissected in situ. One 12-week-old fetus was also transected in the midsagittal plane.

In adult cadavers, the anterolateral abdominal wall was removed through bilateral subcostal and pelvic incisions. The pelvis was isolated by sectioning at the L3–L4 level and below the proximal thigh. Pelvic specimens were sectioned in either the midsagittal or left pararectal sagittal plane. Subsequently, thorough perirectal dissection was performed to highlight the fascial layers enveloping the rectum, as well as the principal neurovascular elements of the pelvis: specifically, the hypogastric plexuses and nerves, and the pelvic splanchnic nerves. Dissection was performed in an unequivocal manner by preserving surrounding elements, such as the main pelvic blood vessels, to offer a clear anatomical context. Additionally, a series of transverse sections were obtained from one male cadaver, enabling observation of the ‘in situ’ disposition of the mesorectal fascia.

We used digital cameras to photograph the dissection pieces. The collection of photographs was further processed digitally using Adobe Illustrator v27 (Adobe Inc., San Jose, CA, USA)to better highlight the structures that we pursued through dissection. No changes were made to the scientific content during this process.

## 3. Results

During fetal life, there is a single dorsal mesentery that connects the abdominal digestive tract to the posterior wall of the torso over its entire length. The dorsal mesentery of the rectum undergoes a complex process of coalescence and involution, resulting in the formation of the mesorectal fascia as an independent structure.

### 3.1. Fetal Mesorectal Development

**Figure 1:**
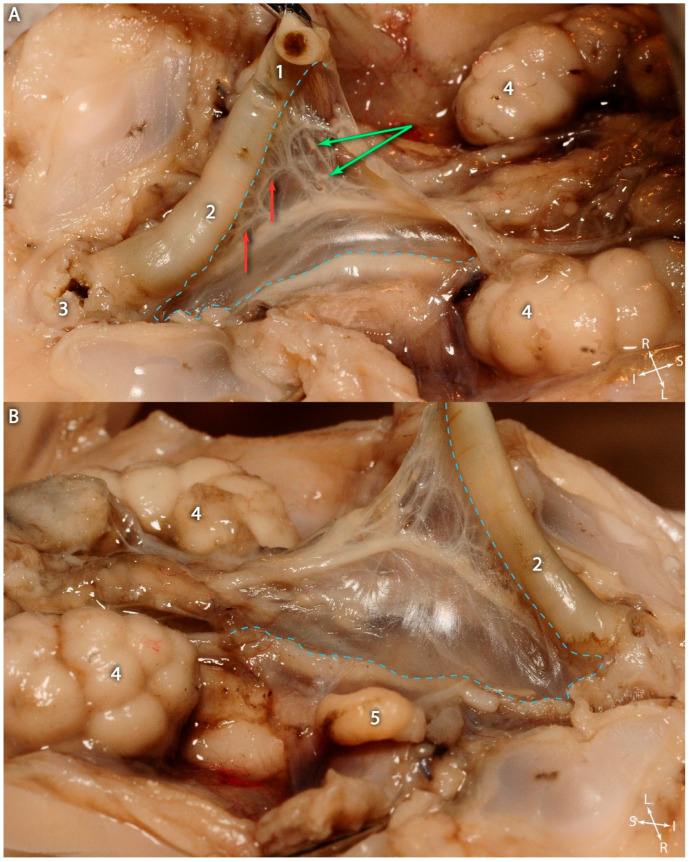
Fetal abdominopelvic cavity—Twelve weeks of gestation. Caudal part of the dorsal mesentery. (**A**). View from the left side. (**B**). View from the right side. 1. Rectosigmoid junction. 2. Rectum. 3. Anal canal. 4. Kidneys. 5. Descending right testis. Green arrows—lymph nodes on the course of the superior rectal artery branches. Red arrows—arterial arches formed by branches of the superior rectal artery. The light blue dotted lines delineate the dorsal mesentery of the rectum. All the photographs include labeled anatomical axes to facilitate spatial orientation: A-anterior, P-posterior, S-superior, I-inferior, R-right, L-left.

In the 12th week, the dorsal rectal mesentery was still present, although it was undergoing a process of involution, as shown in Figure 1A.

The transparency of the dorsal mesentery revealed the superior rectal vessels, from which vascular arches arose in a similar manner to those in the adult mesentery (proper). Lymph node groups could be noted along the course of the vessels.

The involution of the dorsal rectal mesentery occurs progressively from an inferior to a superior direction, as illustrated in Figure 1B. From the dissections, it is readily apparent that the mesorectal fascia was not yet present, and the two peritoneal laminae were still adherent to one another.

**Figure 2:**
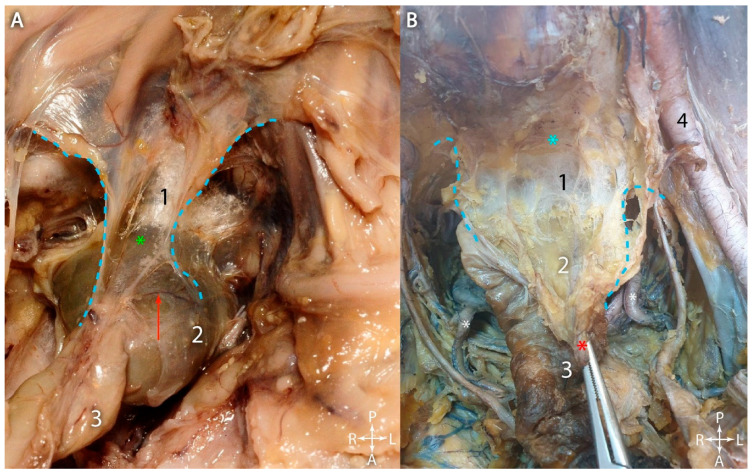
Side-by-side view of the pelvis, viewed from an anterosuperior angle. (**A**). Fetus—fifteen weeks of gestation. (**B**). Adult. 1. “Angel’s hair”. 2. Rectum covered by the mesorectal fascia. 3. Rectosigmoid junction. 4. External iliac artery. Red arrow—superior rectal artery seen through the mesorectal fascia. Green asterisk—mesorectal fascia. Light blue asterisk—sacrum covered by the presacral fascia. Red asterisk—superior rectal artery (resected). Light blue dotted lines—right and left laminae of the former caudal dorsal mesentery.

In the 15-week-old fetus, as shown in Figure 2A, the dorsal mesentery had almost completely disappeared. This is an intermediate situation, much more similar to the condition in adults, as observed in Figure 2B. The right and left laminae of the former dorsal mesentery (light blue dotted line) did not actually coalesce. They spread laterally, generating the pararectal recesses, and the loose connective tissue found between them also extends towards lateral, so that in the adult it lies between the mesorectal fascia and the presacral fascia. This layer of loose connective tissue is used as a cleavage plane during rectal resection with total mesorectal excision, where under antero-posterior traction it appears as thin, translucent connective tissue fibers. Thus, currently it is surgically referred to as “angel’s hair”. Figure 2A depicts the mesorectal fascia, which was transparent and permitted identification of the superior rectal vessels. In adults, as shown in Figure 2B, in the same position as in the fetal dissection, the superior rectal vessels were observed in the clamp, and their course disappeared below the mesorectal fascia. Posterior to this, the “angel’s hair” was observed between the mesorectal fascia and the presacral fascia.

**Figure 3:**
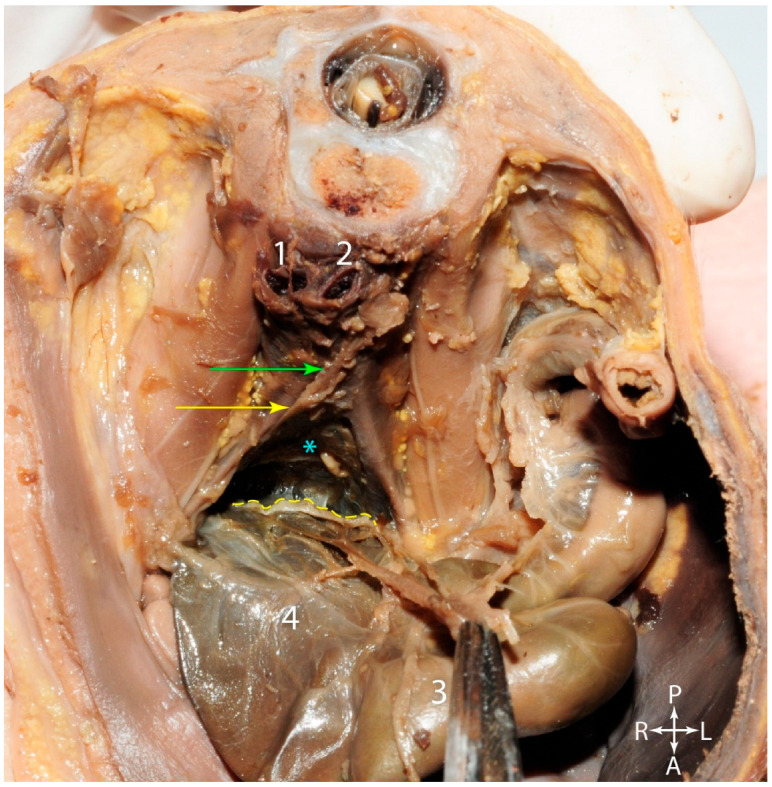
Anterosuperior view of the fetal pelvic cavity (fifteen weeks of gestation). 1. Formation of the inferior vena cava. 2. Caudal part of the aorta. 3. Rectosigmoid junction. 4. Rectum. Green arrow—superior hypogastric plexus. Yellow arrow—right hypogastric nerve. Blue asterisk—anterior aspect of the sacrum. Yellow dotted line—contour of the mesorectal fascia.

As shown in Figure 3, anterior to the bifurcation of the aorta, the superior hypogastric plexus could be observed, as well as the right hypogastric nerve detaching from it to the right. The “Holy Plane of Heald” lies between the mesorectal fascia and the presacral fascia and was highlighted by tractioning the rectum and the mesorectum anteriorly [6]. This maneuver elongated the fibers of loose connective tissue between the mesorectal fascia and the presacral fascia, thus determining the aspect of “angel’s hair” (thin translucent connective fibers, surgically described as ‘angel’s hair’, representing the loose areolar tissue within the natural cleavage plan).

### 3.2. Adult Mesorectal Anatomy

**Figure 4:**
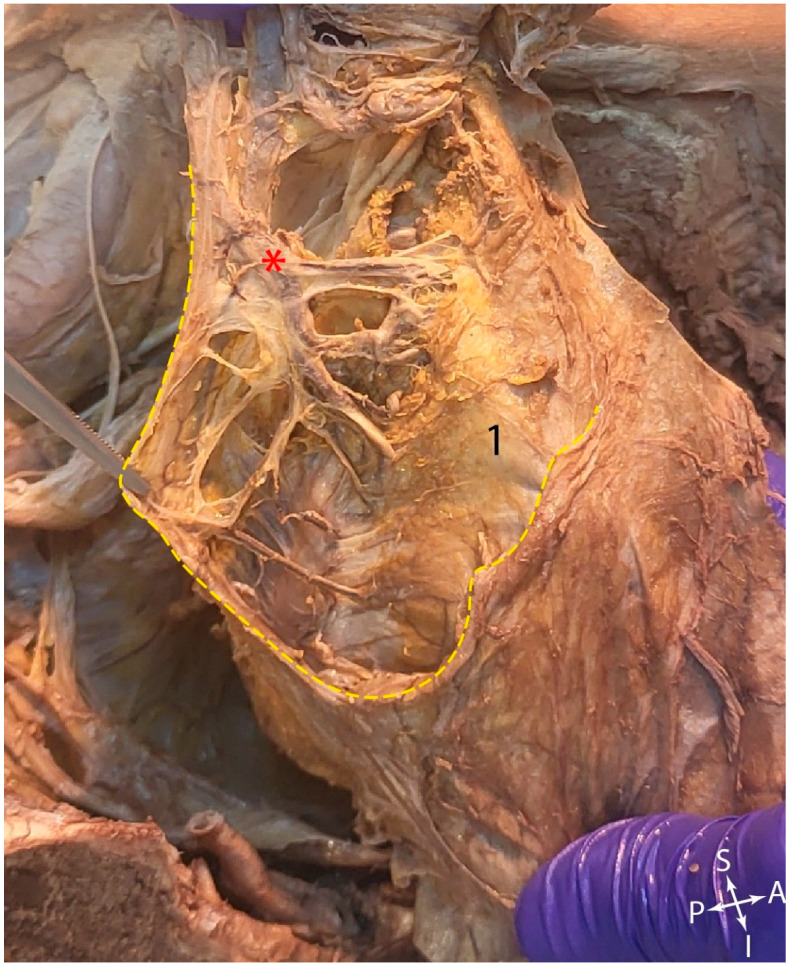
Posterolateral view of the rectum in the adult. 1. Rectum. Red asterisk—superior rectal artery. Yellow dotted line—the mesorectal fascia.

In Figure 3, the forceps anteriorly pulled the superior rectal vascular pedicle, which entered the mesorectal space and was located between the rectum and the mesorectal fascia. The situation was similar to that in adults, as depicted in Figure 4. In the retrorectal space, the mesorectal fascia was held in the forceps, and the superior rectal vessels could be observed as they entered the space between the rectum and the fascia. Notably, there was an abundance of adipose connective tissue, which surrounded the neurovascular and lymphatic elements that coursed through the mesorectal space, as opposed to the fetal mesorectum, which barely contained any adipose connective tissue.

**Figure 5:**
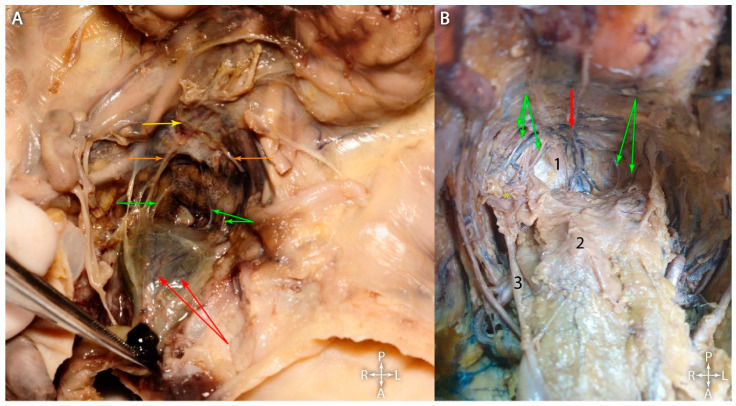
Anterosuperior view of the pelvis. (**A**). Fetus, fifteen weeks of gestation. (**B**). Adult. Yellow arrow—superior hypogastric plexus. Orange arrows—hypogastric nerves. Green arrows—pelvic splanchnic nerves. Red arrow—middle sacral vessels. 1—presacral fascia. 2—mesorectal fascia. 3—right hypogastric nerve, tractioned anteriorly.

In Figure 5A, the rectum was pulled anteriorly and the Holy Plane could be distinguished between the anterior aspect of the sacrum (which has an anterior relationship with the middle sacral artery) and the rectum. The mesorectal fascia was transparent, permitting excellent visualization of the superior rectal vessel branches. On either side of the rectum, the hypogastric nerves could be identified arising from the superior hypogastric plexus. On the right side, the nerve passed lateral to the rectum, between it and the peritoneum. Further down, toward the pelvic floor, the first pair of pelvic splanchnic nerves (parasympathetic nerves that initiate and maintain the erection) were observed on their pathways toward the inferior hypogastric plexuses, to which they supply the parasympathetic component. These nerves were also observed in adults, as shown in Figure 5B, near the pelvic floor. After their emergence from the anterior branches of the sacral nerves, the pelvic splanchnic nerves spread anterior and slightly lateral, advancing through the pelvic subperitoneal connective tissue, towards the inferior hypogastric plexus. This dissection also highlighted the middle sacral artery in a very clear manner, along with its collateral and terminal branches, in relation to the pelvic floor. Furthermore, on the posterior aspect of the rectum, the normal appearance of the mesorectal fascia could be observed. It did not have a regular appearance along its entire length, and surgeons are keenly aware that, in oncological resections for rectal adenocarcinomas, the morphologic integrity of the mesorectal fascia, rather than its regular appearance, is paramount. However, failure to dissect as close as possible to the mesorectal fascia can lead to damage to the nervous structures mentioned earlier.

Even when the fascia appears irregular, maintaining dissection directly along its surface remains essential to avoid inadvertent deviation toward the pelvic autonomic nerves or vascular structures.

The utmost importance is that of the inferior hypogastric plexuses, which received a parasympathetic component from the pelvic parasympathetic nucleus via the pelvic splanchnic nerves. These nerves are responsible for various functions, such as erection and anal and urinary sphincter control. Thus, the loss of their integrity directly affects patients’ quality of life.

**Figure 6:**
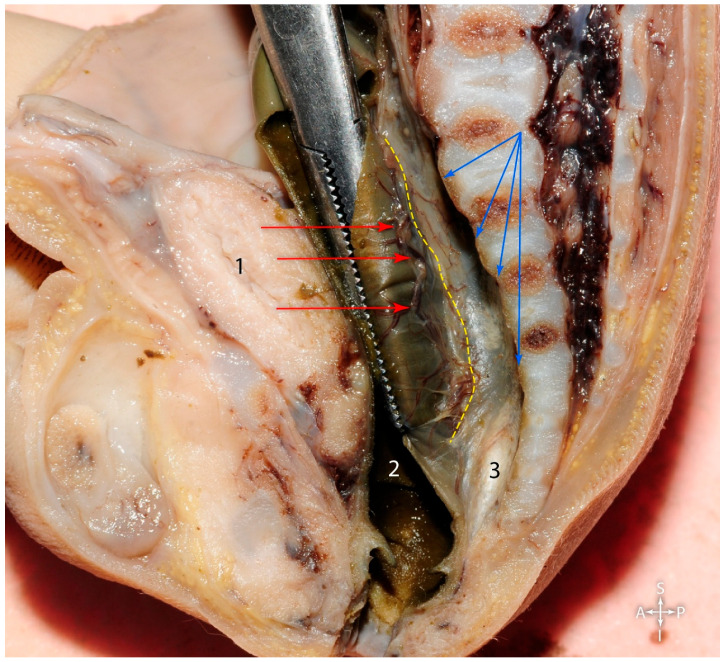
Fetus—twelfth week of gestation, transected along the midsagittal plane. 1. Urinary bladder. 2. Interior aspect of the rectum. 3. “Angel’s hair”. Yellow dotted line—mesorectal fascia. Red arrows—lymph nodes along the branches of the superior rectal artery. Blue arrows—presacral fascia.

In Figure 6, the posterior rectal wall was pulled anterolaterally via forceps, to highlight, within the mesorectal space, the superior rectal artery and the lymph nodes along its course (red arrows). The yellow dotted line indicates the edge of the mediallyresected mesorectal fascia and the blue arrows indicate the presacral fascia. The “angel’s hair” was highlighted posterior to the rectum and anterior to the presacral fascia.

**Figure 7:**
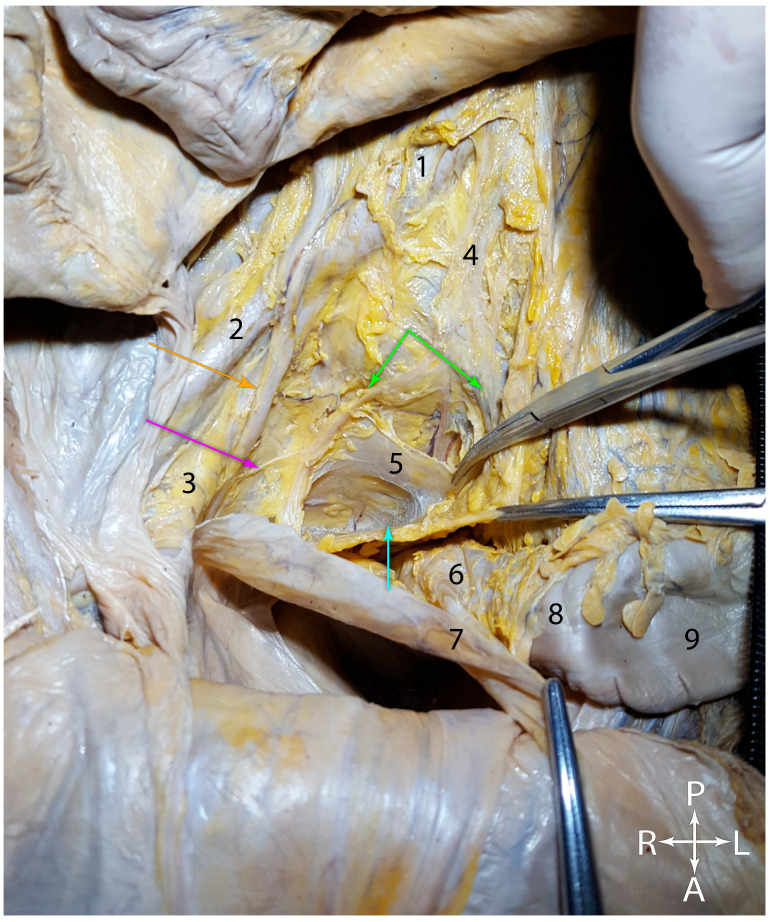
Superior view of the rectum and the mesorectal fascia, in adult cadaver. 1. Right common iliac artery. 2. Right external iliac artery. 3. Right internal iliac artery. 4. Superior hypogastric plexus. 5. Presacral fascia. 6. Rectum inside the mesorectum. 7. Peritoneum reflecting over the anterior aspect of the rectal ampulla. 8. Rectosigmoid junction. 9. Sigmoid colon. Green arrows—right and left hypogastric nerves. Orange arrow—right ureter. Purple arrow—ureteric branch from the right hypogastric nerve. Light blue arrow—“angel’s hair”.

Figure 7 shows a superior view of the rectum and the mesorectal fascia, where the rectum was pulled forward and to the right. For orientation, we noted several landmarks. The upper part of the image shows the space between the two common iliac arteries. The right common iliac artery bifurcation was crossed by the right ureter. Between the two common iliac arteries, the nervous lamina of the superior hypogastric plexus, from which the two hypogastric nerves, right and left, branched off, could be observed with difficulty. Through dissection, the course of the right hypogastric nerve was traced. After giving a ureteral branch, the hypogastric nerve continued its course between the posterior parietal peritoneum of the right pararectal recess and the pelvic wall. The superior rectal vessels were contained in an adipose mass and ran toward the mesorectum. The upper clamp applied tension to a connective tissue lamina that extended between the two hypogastric nerves, the middle clamp held the mesorectal fascia, and the lower clamp retracted the peritoneum of the right pararectal recess.

**Figure 8:**
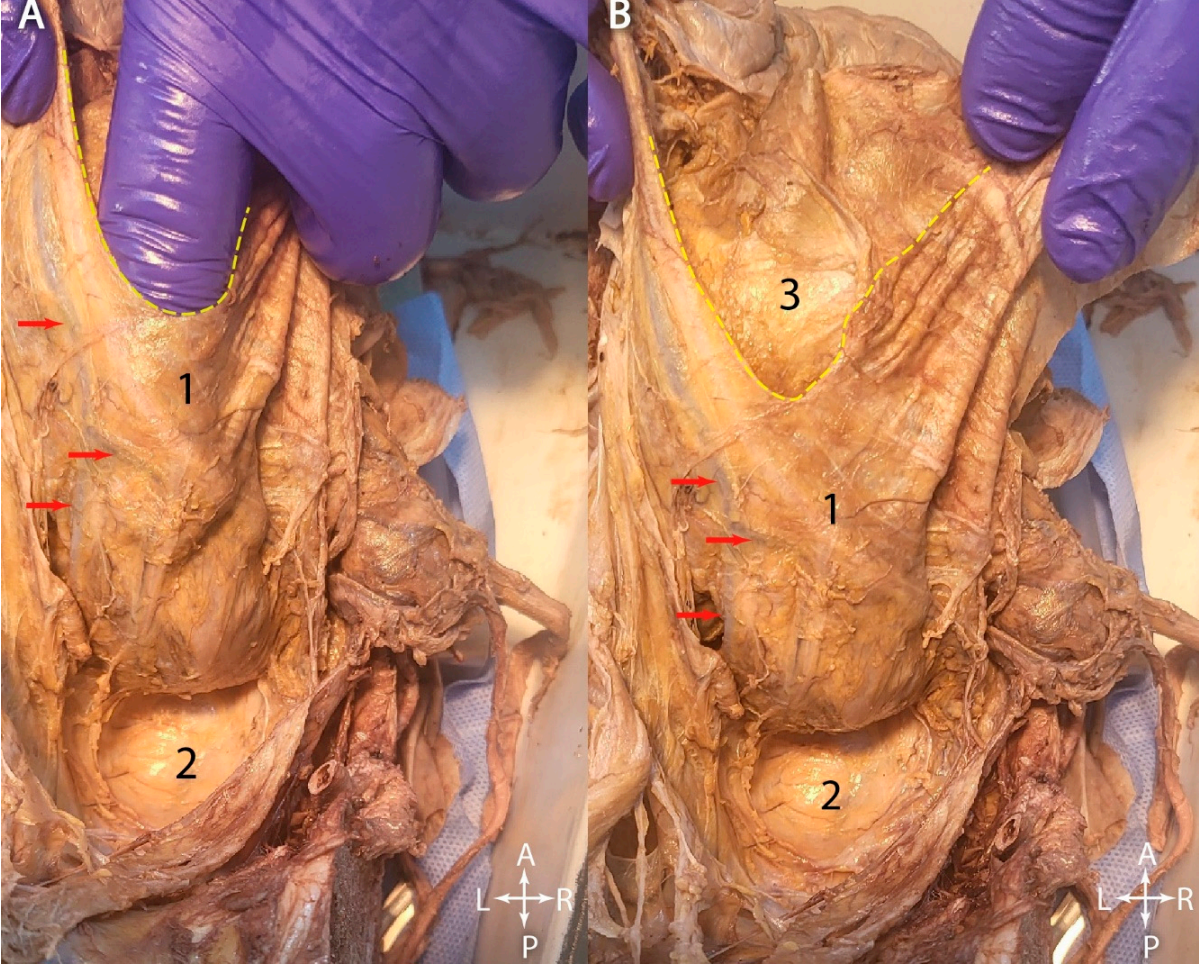
Posterior aspect of the rectum covered by the mesorectal fascia. (**A**) Operator’s index highlighting the mesorectal space. (**B**) Mesorectal fascia in situ. 1. Rectum (covered by the mesorectal fascia). 2. Sacrum covered by the presacral fascia. 3. Mesorectal adipose connective tissue surrounding the rectum. Yellow dotted line—contour of the mesorectal fascia. Red arrows—branches of the superior rectal vessels, branching inside the mesorectal space.

Figure 8A shows the operator’s index finger inserted between the rectum and the mesorectal fascia. Through its transparency, the superior rectal vessels could be seen inside the mesorectum. The Holy plane could be very well visualized, between the anterior aspect of the sacrum and the mesorectal fascia. Despite the amount of mesorectal adipose connective tissue, the branches of the superior rectal vessels could be observed through the transparent mesorectal fascia, which demonstrated their superficial course proximally. Hence, if TME is not properly performed, and the mesorectal fascia is discontinued during dissection, there is also a risk of hemorrhage. Figure 8B highlights the superior limit of the mesorectal fascia.

**Figure 9:**
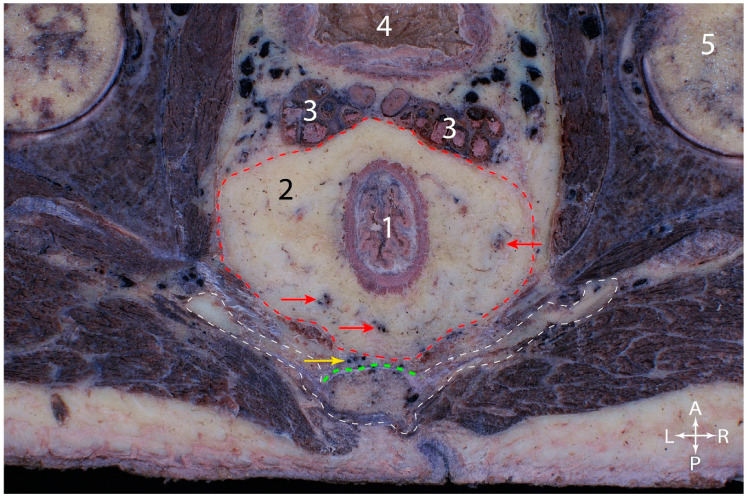
Transverse section of the male adult pelvis. 1. Rectum. 2. Mesorectal adipose connective tissue. 3. Seminal vesicles. 4. Urinary bladder. 5. Right femoral head. Red dotted line—contour of the mesorectal fascia. Green dotted line—presacral fascia. White dotted line: contour of the sacrum. Red arrows—branches of the superior rectal vessels. Yellow arrow—middle sacral vessels.

In Figure 9, on a cross-section through the male pelvis, at the level of the seminal vesicles, the rectum, surrounded by the mesorectum, with abundant adipose connective tissue and branches of the superior rectal artery, could be identified in the center. The mesorectum was enveloped by the mesorectal fascia, indicated by the red dotted line. Posterior to the mesorectal fascia was the presacral fascia (green), superficial to which the middle sacral vessels descended. Between the two fasciae is the “Holy plane”.

### 3.3. Comparative and Surgical Anatomical Correlations

The comparative analysis of fetal and adult specimens demonstrates a clear continuity between the developmental remodeling of the dorsal rectal mesentery and the configuration of the mesorectal compartment observed in adulthood. The progressive lateral divergence of the fetal mesenteric laminae corresponds topographically to the loose connective tissue plane identified between the mesorectal and presacral fasciae in adult dissections. This anatomical continuity provides a morphological explanation for the surgically recognized avascular plane described during total mesorectal excision. The delicate areolar connective tissue fibers, referred to intraoperatively as “angel’s hair,” were consistently observed as a transitional layer between these fascial boundaries, reinforcing their developmental origin rather than a surgically created artifact. Furthermore, the spatial relationships between the mesorectal fascia and the hypogastric and pelvic splanchnic nerves appeared conserved from fetal development to adult anatomy, highlighting the close proximity of autonomic neural elements to the lateral and posterior mesorectal boundaries. These observations emphasize how embryological anatomy may help explain the organization of adult surgical planes, while the present study remains limited to morphological description without assessment of clinical outcomes or operative performance.

## 4. Discussion

Classical anatomical descriptions were intentionally included to reflect the historical evolution of mesorectal concepts, complemented by contemporary surgical and imaging literature.

The mesorectal complex develops after formation of the rectum, during a critical window, between the 12th and 15th weeks of gestation [9]. Unlike the colonic mesentery which undergoes the process of coalescence, partly generating the Toldt’s fascia, the dorsal mesentery of the rectum undergoes a process of lateral divergence and condensation. This aspect carries a significant importance, because a simple coalescence would result in a layer of loose connective tissue that would not present a clear, definitive limit. Instead, the evolution of the rectal dorsal mesentery creates a structure that is enclosed in a newly developed fascia. In early gestation, this mesentery comprises paired peritoneal laminae extending from the posterior abdominal wall to the rectum. As development progresses, these laminae gradually diverge laterally, and the interposed connective tissue evolves into a loose areolar layer [10]. The fascial planes established by week 15 of gestation remain morphologically stable throughout further maturation.

In adult anatomy, this connective tissue corresponds to the plane between the mesorectal fascia and the presacral fascia [6,11]. The mesorectal fascia forms a continuous envelope around the rectum and its associated vascular, neural and lymphatic structures, defining the mesorectal compartment. Posterior to this fascia, the presacral fascia covers the anterior surface of the sacrum [12]. The persistent plane between these two fasciae constitutes the natural cleavage plane encountered during posterior rectal dissection, called the Angel’s Hair [6,11,13]. From a practical standpoint, the appearance of the delicate connective fibers described as ‘angel’s hair’ may serve as an intraoperative visual cue indicating correct entry into the natural avascular plane, thereby assisting surgeons in maintaining the integrity of the mesorectal envelope.

Although Denonvilliers’ fascia represents an important anterior pelvic structure, the present dissections were primarily oriented toward the posterior mesorectal and presacral planes; therefore, consistent visualization suitable for photographic documentation was not obtained.

During this morphogenic process, as the rectum grows, the superior rectal vessels descend beneath the developing mesorectal fascia [9]. Throughout this evolution, the superior rectal vessels adopt a branching pattern reminiscent of the superior mesenteric artery, giving rise to pararectal arterial arches and terminal straight branches. Lymph nodes are consistently observed along the trajectory of the arterial arches, reflecting the involvement of the mesorectum in the lymphatic drainage of the rectum [14,15]. The resulting pararectal recesses effectively demarcate the lateral limits of the mesorectum and help define the “Holy plane”, a crucial anatomical and surgical landmark that facilitates precise, nerve-sparing dissection during total mesorectal excision [6,13].

From a surgical perspective, the mesorectum encloses vital vascular and lymphatic structures, while running in close anatomical proximity to key autonomic nerves, including the hypogastric nerves, which descend outside its fascial boundaries, along the pelvic sidewall [16]. These nerves are integral to the sympathetic innervation of the pelvic viscera and emerge from the inferior portion of the superior hypogastric plexus near the sacral promontory. The hypogastric nerves then give rise to the inferior hypogastric plexuses, which play a key role in the innervation of the urinary bladder, prostate, and internal genitalia, as well as the rectum [17]. Injury to the hypogastric nerves and hypogastric plexuses can result in loss of function of the urinary and anal sphincters, retrograde ejaculation, and loss of orgasmic sensation [18]. Their preservation is essential for maintaining urinary continence and sexual function, particularly in male patients [19,20]. Precise identification and protection of these nerves are therefore essential in nerve-sparing rectal resection with TME [18,21].

Below the hypogastric nerves lie the pelvic splanchnic nerves (S2–S4), which provide parasympathetic innervation to the urinary bladder, genital viscera, and rectum and are responsible for detrusor muscle contraction and sexual function [17,20]. These nerves converge into the inferior hypogastric plexus near the pelvic floor and lie adjacent to the lateral ligaments of the rectum, often enveloped in loose connective tissue, along with small vascular branches [16]. They are particularly vulnerable in the lower pelvic dissections. Injury to these fibers may result in urinary retention, erectile dysfunction, anejaculation, or impaired vaginal lubrication [20,22,23]. Therefore, precise anatomical knowledge of their course is paramount for performing nerve-sparing dissections and achieving a proper postoperative quality of life.

Technological advances, particularly in laparoscopic and robotic-assisted TME, have significantly improved intraoperative visualization, enabling more refined and consistent nerve-sparing techniques [24]. These minimally invasive approaches offer equivalent oncological outcomes to open surgery, while lowering rates of postoperative genitourinary dysfunction [25]. Robotic systems, in particular, may allow for enhanced precision and three-dimensional visualization in confined pelvic spaces, although the superiority of robotic surgery has yet to be demonstrated. However, given the complex three-dimensional nature of the mesorectum, the capacities of advanced robotic and laparoscopic systems are highly useful during TME surgery [26,27].

International practice patterns reflect differing strategies for nerve preservation. In Japan, for example, lateral lymph node dissection has long been a standard adjunct to TME for low rectal cancer, although it poses a high risk to the pelvic autonomic nerves. More recently, randomized studies have demonstrated that, when neoadjuvant therapy is used, nerve-sparing TME without lateral node dissection achieves comparable oncological outcomes and significantly better functional preservation [28].

Finally, the etymology of “mesorectum” warrants clarification [29]. The term is not derived from direct anatomical continuity with the mesentery of the small bowel. Instead, it originates from the Greek “mesos” (“middle”), referring to the compartment between the rectum and its fascial envelope [30]. This region contains the essential neurovascular and lymphatic components of the rectum and constitutes a decisive anatomical landmark in pelvic surgery.

In summary, the mesorectum represents a complex embryological and anatomical entity, developed after the remodeling of the dorsal rectal mesentery, whose understanding is central to modern colorectal surgery. Careful identification of the mesorectal boundaries, vascular elements, and autonomic nerves provides anatomical insights relevant to safe and nerve-aware surgical strategies, although the present study does not assess clinical outcomes.

### Limitations

This study has several limitations that should be acknowledged. The number of fetal specimens was limited, reflecting the rarity of available material, and all adult tissues were formalin-fixed cadaveric specimens, which may differ from in vivo conditions. Nevertheless, the principal anatomical relationships were consistently observed across all examined specimens, supporting the reliability of the described findings. Future imaging-based or clinical studies are required to further explore the translational relevance of these anatomical observations. A key limitation of this work is the absence of clinical correlation; therefore, any surgical implications should be interpreted as anatomically grounded hypotheses rather than demonstrated therapeutic advantages.

Future prospective surgical or imaging-correlated studies are required to evaluate whether these anatomical observations translate into measurable oncologic or functional benefits.

## 5. Conclusions

Our findings demonstrate that the mesorectal fascia represents a pre-determined developmental entity that forms during the fetal development, observable between the twelfth–fifteenth weeks of gestation, through the lateral divergence of the dorsal mesentery. This process establishes the mesorectal fascia as a definitive anatomical envelope, containing the superior rectal vessels and lymph nodes, which is consistently identifiable as a distinct connective tissue layer. Our findings support the concept that the ‘Holy plane’ reflects an anatomically pre-existing fascial relationship established during development, rather than solely a surgically created space. No consistent sex-related differences were observed in the configuration or fascial relationships of the mesorectal structures in the examined specimens.

The identification of the loose connective tissue plane (“Angel’s hair”) between the mesorectal and presacral fasciae is essential for successful total mesorectal excision. Adherence to this embryologically defined plane may help preserve the integrity of the mesorectal compartment and provides a safe distance from the hypogastric and pelvic splanchnic nerves. Consequently, a comprehensive understanding of the embryological evolution and morphological relationships of the mesorectum provides surgeons with an invaluable anatomical landmark. The identification of the embryologically defined mesorectal planes and associated neurovascular relationships provides a detailed anatomical framework relevant to modern rectal surgery. While these observations may have implications for surgical plane selection and nerve preservation, the present study remains purely anatomical and does not evaluate operative safety, oncological margins, or functional outcomes. Future clinical and imaging-based investigations are required to determine the translational impact of these findings.

## Data Availability

All data generated and analyzed during the current study are included in this published article.

## Abbreviations

The following abbreviations are used in this manuscript:

TME	Total Mesorectal Excision
